# Biocellulose for Incisional Hernia Repair—An Experimental Pilot Study

**DOI:** 10.3390/nano9020236

**Published:** 2019-02-10

**Authors:** Falk Rauchfuß, Julian Helble, Johanna Bruns, Olaf Dirsch, Uta Dahmen, Michael Ardelt, Utz Settmacher, Hubert Scheuerlein

**Affiliations:** 1 Department of General, Visceral and Vascular Surgery, Jena University Hospital, 07747 Jena, Germany; julian.helble@gmx.de (J.H.); Johanna.Bruns@med.uni-jena.de (J.B.); Michael.Ardelt@med.uni-jena.de (M.A.); Utz.Settmacher@med.uni-jena.de (U.S.); H.Scheuerlein@vincenz.de (H.S.); 2 Experimental Transplantation Surgery, Department of General, Visceral and Vascular Surgery, Jena University Hospital, 07747 Jena, Germany; Olaf.Dirsch@med.uni-jena.de (O.D.); Uta.Dahmen@med.uni-jena.de (U.D.); 3 Institute of Pathology Hospital of Chemnitz, 09116 Chemnitz, Germany; 4 Department of General and Visceral Surgery, St. Vinzenz Hospital, 33098 Paderborn, Germany

**Keywords:** mesh, mesh infection, abdominal wall, reconstruction

## Abstract

Ventral or incisional hernia are a common disease pattern in general surgery. Most commonly, a mesh repair is used for reconstruction, whereby the mesh itself might cause complications, like infections or adhesions. Biological materials, like biocellulose, might reduce these clinical problems substantially. In this prospective rodent study, a biocellulose mesh (produced by Gluconacetobacter xylinus) was implanted either by a sublay technique or as supplementation of the abdominal wall. After an observation period of 90 days, animals were sacrificed. The adhesions after the reconstruction of the abdominal wall were moderate. The histologic investigations revealed that the biocellulose itself was inert, with a minimal regenerative response surrounding the mesh. The explanted mesh showed a minimal shrinkage (around 15%) as well as a minimal loss of tear-out force, which might be without clinical relevance. This is the first in vivo study describing biocellulose as a suitable mesh for the repair of ventral hernia in two different hernia models. The material seems to be a promising option for solving actual problems in modern hernia surgery.

## 1. Introduction

The rate of incisional hernia after a laparotomy is up to 20% [1,2,3]. Therefore, it is a large economic burden, especially if a hernia recurrence occurs.

In most guidelines, a reconstruction of the abdominal wall using a mesh is recommended to decrease the risk of recurrence [4]. The operation technique is determined depending on the localization, size, and previous operations: the laparoscopic approach requires a mesh implantation, whereby the use of a mesh in the open operations method depends on the size of the defect of the abdominal wall. The most common techniques for mesh implantation are the sublay method (where the mesh is placed retromuscularly in the rectus sheath) and the complete intraperitoneal reparation of a defect of the abdominal wall.

Most meshes are synthetic, the market for which is multitudinous. An ideal mesh should reconstruct the function of the abdominal wall, should be integrated in the surrounding tissue with maximum biocompatibility, should not cause any adhesions or fistula, can be easily handled, and should have the lowest possible risk of mesh infections [5]. The latter is a greatly feared complication, as the mesh must be removed completely and the abdominal wall defect is subsequently recurrent in most cases. In recent years, so-called “biological meshes” are used for abdominoplasty, if a synthetic mesh had to be explanted and a mesh reconstruction is necessary in an infected area. Such biological meshes are mostly human, bovine or porcine dermis, bovine pericardium, or porcine submucosa of the small intestine [6]. However, biological meshes are expensive. Nevertheless, the field of biological meshes is the focus of research activities all around the world.

The actual challenges in hernia research are: long-term biocompatibility, mechanical stability, less possible shrinkage of the mesh and seroma formation, resistance against infections, avoidance of adhesions and fistula, and a lowest possible rate of hernia recurrence.

The ideal mesh fulfilling all of the mentioned requirements hasn’t been found yet. 

Biocellulose (BC) is a naturally occurring homopolymer of glucose. It is water-insoluble and it is limited when cleaved in human or animal organisms, since specific hydrolases are missing. The main properties which are interesting and important for potential clinical use are: purity, in situ formability, high degree of polymerization and crystallinity, large inner surface due to a network of nanofibers, distinct mechanical stability, and tensile as well as tear strength. Therefore, biocellulose has been applied in several areas of medicine including duraplasty, wound dressing, and the reconstruction of tympanic perforations [7].

The aim of this study was the evaluation of biocellulose in two different models of hernia surgery, on the one hand as mesh in sublay hernia reconstruction and on the other hand as complete abdominal wall reconstruction.

## 2. Methods

### 2.1. Manufacturing of the BC Patches

The manufacturing process was described previously [8]. In brief, BC was fabricated in the form of patches by using Gluconacetobacter xylinus bacteria. Twenty volume parts of Hestrin-Schramm medium were inoculated with 1 volume part of a 7–day-old liquid preculture. A six-well plate was filled with this culture (2.5 mL/well), which was then cultivated at 28 °C under static conditions for 14 days. The BC fleeces were taken from the culture medium, washed with distilled water, treated with boiling 0.1 N aqueous sodium hydroxide for 10 min, and washed with distilled water to a neutral reaction of the rinsing agent. The fabricated never-dried fleeces had a diameter of 35 mm and a thickness of either 1.0–1.5 mm (sublay arm) or 1.5–2.0 mm (abdominal wall replacement). The nanofibrilles had a diameter between 20–100 nm. The material was steam sterilized (1 bar, 120 °C) for 20 min and stored in Ringer’s solution until required for use.

### 2.2. Experimental Design

We used 20 male Wistar rats with an age of 12 weeks and a median weight of 300 g in this prospective animal experiment. The experimental design was approved by the Thüringer Landesamt für Lebensmittelsicherheit und Verbraucherschutz (Registration number 02-020/11). The animals were delivered by Charles River Laboratories (Sulzfeld, Germany). The rats were kept in the Institute for Experimental Surgery of the Friedrich-Schiller-University, Jena, Germany. The operation was performed after an acclimatization period of at least seven days after delivery.

Two groups were built: one group (*n* = 10) with the BC in sublay position and the other group (*n* = 10) using the BC as abdominal wall replacement.

The experimental design was as follows:

All operations were performed with inhalation anesthesia (isoflurane).

### 2.3. Sublay Technique:

**Figure 1:**
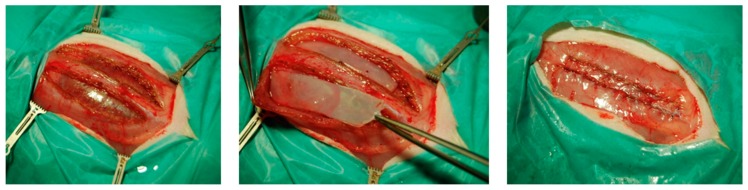
Intraoperative images of the sublay model: **Left**—Preparation of both rectus sheaths. **Middle**—Placement of the biocellulose patches. **Right**—Fascia closure.

A median skin incision was performed and the subcutaneous layer was mobilized up to the lateral edges of the rectus sheath. The latter one was opened on both sides approximately 3 millimeters away from the *linea alba* at a length of 45 millimeters. The rectus muscle was mobilized to create a preperitoneal compartment where the BC (with a dimension of 4 × 1 centimeters) was placed, see Figure 1. After proper placement, the rectus sheath was closed, using a running suture MOPYLEN® 6/0 USP (Fa. Resorba, Nuremburg, Germany). Skin was closed using a running resorbable suture.

### 2.4. Abdominal Wall Replacement:

**Figure 2:**
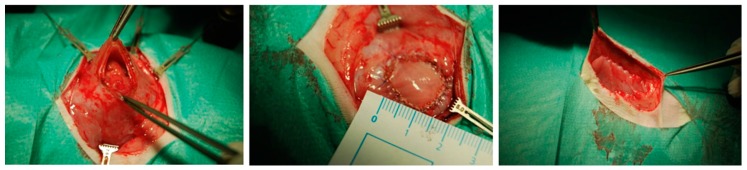
Intraoperative images of the abdominal wall replacement model: **Left**—Preparation of the abdominal wall defect. **Middle**—Defect closure with the biocellulose patch. **Right**—Situs before closure of the subcutaneous tissue.

The skin incision was performed 2 cm right from the median line. The subcutaneous layer was mobilized, and then retracted to the left side to get an almost complete exposition of the ventral abdominal wall. A circular (2 cm diameter) area was marked and excised including the fascia, muscles and peritoneum. After that, the resulting defect was reconstructed using an appropriate BC patch (see Figure 2), which was fixed with four interrupted sutures, followed by a running fixation suture (MOPYLEN® 6/0 USP [Fa. Resorba, Nuremburg, Germany]). Skin was closed using a running resorbable suture.

The postoperative follow-up period was 90 days. 

After 10, 31, and 90 days, ultrasound investigations were performed to explore eventual seroma or perigraft reactions. Furthermore, the thickness of the different layers of the abdominal wall was measured.

After the final ultrasound examination, the animals were anesthetized and a relaparotomy was performed. Particularly in the group of the abdominal wall replacement, the abdominal cavity was opened as laterally as possible to assess potential adhesions to the patch in the complete area. For the evaluation of the adhesions, a slightly modified Vandendael score was used [9]. The score is subdivided in four different criteria (extent of the adhesion, thickness, strength, and adhesion formation beyond the mesh) with a scoring between 0 and 3. Therefore, a score of “0” means “no adhesions” and a score of “12” means “very severe adhesions”.

The additional macroscopic evaluation included the judgement of tissue integration, seroma, and local inflammation reactions. Furthermore, the implanted patch was measured precisely to judge potential shrinkage processes. After the harvesting of tissue samples (for histologic, immunohistochemical analysis, and tension testing), the animals were sacrificed by exsanguination.

### 2.5. Histologic Examinations

The tissue was fixed in formalin and subsequently paraffine-embedded. For further analysis, we used sections measuring 4 μm.

We used hematoxylin-eosin staining for the judgement of tissue integration and material incorporation, periodic acid-Schiff reactions for the exclusion of fungal infections, Elastica-van-Gieson staining for the quantification of histiocytic giant cells and scar tissue, Naphthol-AS-D-Chloracetatesterase (ASDCL) for the quantification of granulocyte infiltration, and Bromdesoxyuridin (BrdU) staining for the assessment of the proliferative activity within the tissue per 0.3 mm^2^. All stainings were performed according to a standardized protocol.

### 2.6. Tensile Testing 

For the tests, a representative tissue probe, including a standardized proportion of the BC patch and the surrounding tissue, was natively frozen. The testing itself was performed in close collaboration with the Institute of Textile Machinery and High Performance Material Technology at the Technical University in Dresden, Germany.

The device used was the tensile testing machine Zmart Pro (Zwick GmbH&Co. KG, Ulm, Germany). Prior the testing of the experimental probes, native BC patches were also frozen and subsequently tested, and the obtained values were used for comparison with the experimental data. In brief, the probes were clamped in the machine and the jaws of the machine diverged with a standardized speed of 10 mm/min. The integrated Zmart Pro software (Zwick GmbH&Co. KG, Ulm/Germany) measured the tissue tension (in mm) against the raised force (in N). The experiment was performed up to reaching the wrest point of the material.

### 2.7. Statistical Analysis

Statistical analysis was performed using SPSS, Version 20.0 (IBM Company, Armonk, NY, USA). The median of all values and the standard deviation was calculated. Between both groups and for comparison with native BC values (tensile testing), the Mann-Whitney U test was used for independent values and the Wilcoxon test for dependent values. A *p*-value of < 0.05 was considered to be significant.

## 3. Results

### 3.1. General Data

All animals recovered well from the operation. After the usual weight loss directly after the operation, the animals showed a steady weight gain. In some individuals, a seroma had to be diagnosed. All animals recovered throughout the observation period.

### 3.2. Adhesions

**Figure 3:**
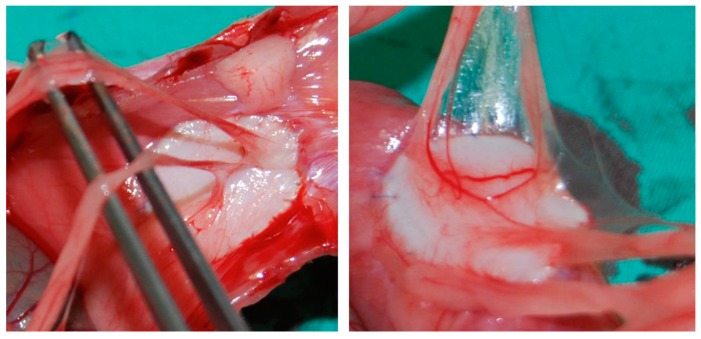
(Partly extensive) adhesions after relaparotomy in the abdominal wall replacement model. Both Figures show different adhesions after the abdominal wall replacement.

In the group receiving a sublay reconstruction, there were no intraabdominal adhesions, which lies in the nature of the applied methodology, since the abdominal cavity was not opened. As opposed to this, adhesions in the group of animals which had undergone an abdominal wall replacement were obvious. Nevertheless, the modified Vandendael score showed interindividual variations with values between 0 and 10 (median value: 6), see also Figure 3. 

### 3.3. Material Shrinkage

#### 3.3.1. Sublay Technique

In this group, the BC patch showed an average shrinkage of 15.38 ± 9.72%. However, there was interindividual variation with a maximum shrinkage of 37.9% and a minimal shrinkage of 2.6%.

#### 3.3.2. Abdominal Wall Replacement

Since the patch was spherical, the shrinkage was measured in two dimensions, longitudinally and oblong. The median shrinkage in both dimensions was 10%. Therefore, the median shrinkage of the area was around 17%.

### 3.4. Histologic Examinations

#### 3.4.1. Hematoxylin-Eosin Staining and Elastica-van-Gieson Staining

**Figure 4:**
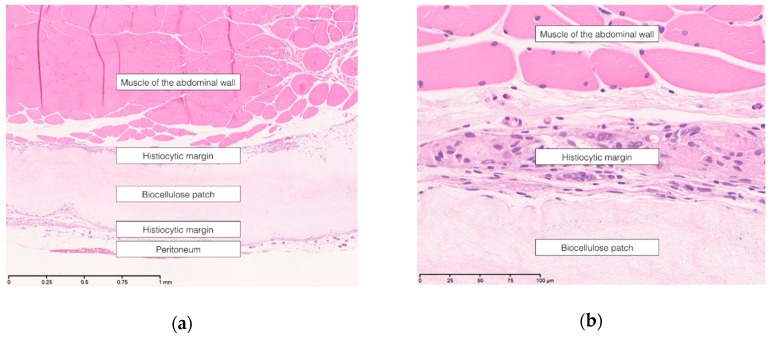
Hematoxylin-eosin staining showing the histiocytic margin and the patch in the sublay model, (**a**) 100-fold magnification; (**b**) 200-fold magnification.

The biocellulose patch was supposed to be an inert material in both groups. There were no histiocytic infiltrations, except in the overlap area between patch and surrounding tissue (see Figure 4a,b).

This was confirmed by the Elastica-van-Gieson staining.

#### 3.4.2. Naphthol-AS-D-Chloracetatesterase (ASDCL) Staining

There was no indication of an active inflammation reaction in the sublay group nor in the group of abdominal wall replacement.

#### 3.4.3. Periodic Acid-Schiff Reaction

**Figure 5:**
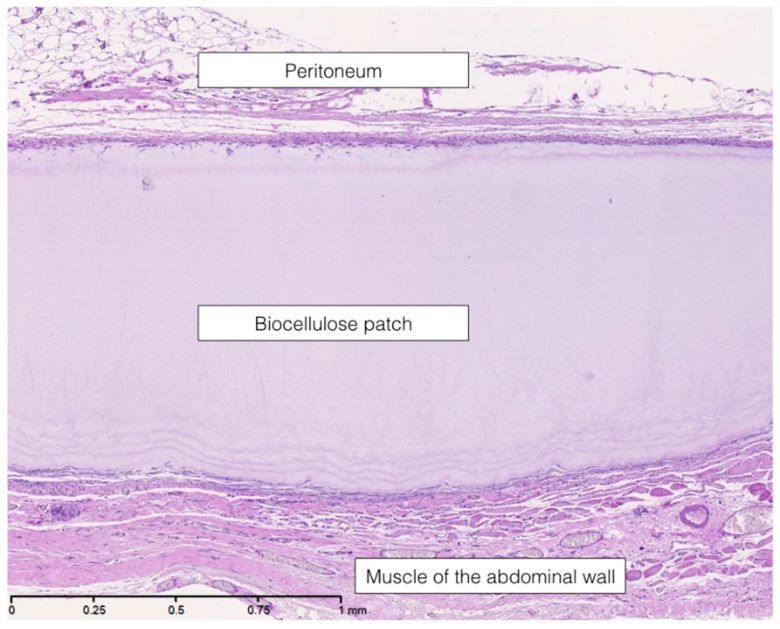
Periodic acid-Schiff reaction in the sublay model, 100-fold magnification.

In none of the groups did the periodic acid-Schiff reaction reveal hints of a fungal infection (see Figure 5).

#### 3.4.4. Bromdesoxyuridin (BrdU) Staining

The BrdU staining showed a difference in BrdU-positive cells, depending on the localization within the patch. Near the end of the patches a median of 6.25 BrdU-positive cells could be detected, whereas in the middle of the patch, only a median of 2.5 BrdU-positive cells could be counted. This could mean that areas with more mechanical stress (the end of the patch) have an increased proliferation.

### 3.5. Tensile Testing

In the native patches, the force needed to break the material was 5.54 N ± 2.44 N for the biocellulose used for the sublay reconstruction, and 9.36 N ± 1.21 N for the patches in the abdominal wall replacement setting. 

**Figure 6:**
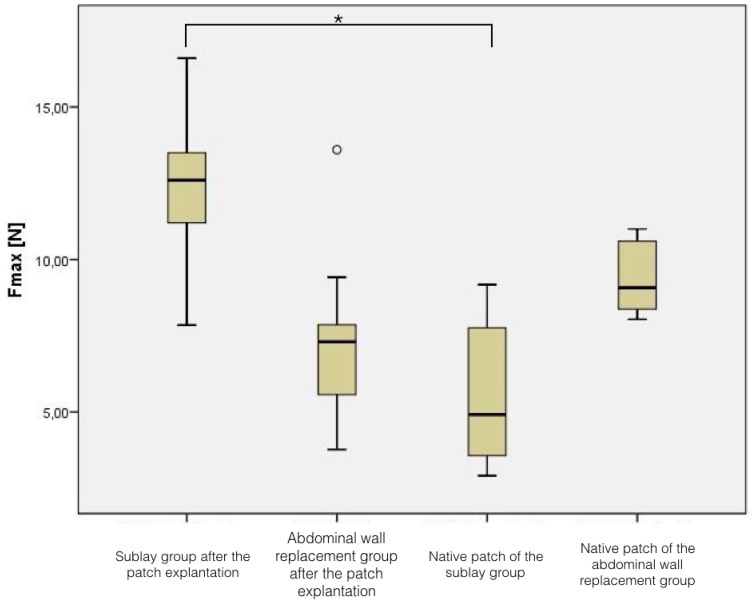
Tensile testing results depending on the group affiliation.

After the implantation in the sublay technique, the required force for breaking the material was 12.47 N ± 2.21 N, reflecting an increase of breaking force of 125%, (*p* < 0.001 compared to the native material). In contrast, the patches which were used for abdominal wall replacement showed a decrease of breaking force of 23%, since the force needed was 7.22 N ± 2.97 N (*p* = 0.05 compared to the native material), see Figure 6.

## 4. Discussion

The aim of the study was a proof-of-concept as to whether biocellulose is suitable mesh in common hernia models. The standard of care in the treatment of hernia patients is the use of a mesh in the majority of cases. This leads to specific complications, like mesh infections or fistula, which are difficult to treat [10].

Surgeons all around the world are searching for meshes which are biocompatible, to avoid or at least decrease the risk of infectious complications. Indeed, there are several providers with biological meshes on the market. These materials have mostly a porcine or bovine origin. The main disadvantage is the high costs which go along with these biological meshes. Therefore, the meshes are mostly applied in the infectious situs after the explantation of an infected conventional mesh [11].

To avoid an infection a priori, hernia surgeons aim for well-tolerated meshes without an infectious potential, which is easily produced and not expensive. Biocellulose might be such a material. It combines important cellulose properties with the features of nanomaterials. These properties are, in particular, high purity (pure cellulose), a nanofiber network structure, and a high water content of 99%, in the form of mechanically and thermally stable hydrogel bodies [7]. Furthermore, it is chemically inert, mechanically stable, and can be sterilized. This led to different medical applications, some already being used in clinical settings, like wound dressings [12] or dura meshes [13], and some are experimental, either in patch form like this described approach, or in tube form, for uses such as blood vessel bypass [14,15].

For the evaluation of biocellulose in these experiments, we have chosen two different models to simulate the clinical reality as precisely as possible: the sublay technique in rodent models was described by Tanaka et al. [16], and clinically, reflects the most frequently applied technique in open hernia repair. In an experimental setting, one must take care that rats don’t have a prominent posterior rectus sheath. Therefore, the preparation has to be undertaken carefully to avoid defects of the peritoneum.

Several workgroups described the second used model [9,17], the abdominal wall replacement, which simulates intraperitoneal online mesh (IPOM), a method which is used either laparoscopically or in open surgery.

There is only one experimental workgroup dealing with the issue of using biocellulose as an abdominal wall replacement [18,19]. The authors subdivided 60 animals in two groups comparing biocellulose vs. ePTFE-membranes. Endpoints were the incorporation measures and biomechanical properties. Even if this study is the first dealing with this question, there are several points which have remained controversial: first, Falcao’s group used Zoogloea species for the biocellulose synthesis, whereas the most experiments worldwide are done with biocellulose synthesized by Acetobacter xylinus. Second, the material was (air-)dried and partly compressed, which affects the mechanical stability of the material [20]. Third, the material was fixed with large suture distances (compared to the size of the defect). Fourth, the used PTFE-probes were taken from vascular protheses with a pore diameter of 25 μm (meshes for abdominal wall replacement usually have a pore diameter of 3–22 μm). Since the pore diameter is an important factor for the incorporation of the material, this might affect the results. Furthermore, the pore diameter of patches synthesized by Zoogloea species is 0.07 μm, the pore size after Acetobacter xylinus synthesis is 10 μm. For the stated reasons, it is nearly impossible to compare the studies by Falcao et al. with our results.

One major issue in hernia repair with meshes is the risk of mesh shrinkage. This was also a phenomenon in our study. Compared to well-defined PTFE material, biocellulose also showed a tendency to shrink. This phenomenon is already known in biocellulose, and even in modifications of biocellulose [21,22]. It might be a phenomenon of cellulose degradation, which could be caused by the sterilization process. How far this might be a clinical problem remains unclear, and should be investigated in further (long-term) studies.

All patches were macroscopically well-integrated without differences between the groups. At the end of the experiments, there were no seroma or local signs of infections.

To analyze the standardized adhesions, we used the well-established (modified) Vandendael score [23]. Naturally, adhesions play the major role in the abdominal wall replacement. However, compared to the well-known IPOM meshes, which are extensively described in the literature, the adhesions in the abdominal wall replacement group were less marked. The formation of adhesions is well studied, both in human [24] and in animal studies [25]. It is still a matter of debate, if and how meshes should be modified (e.g., barrier gels). This could be even a further option of BC modification to decrease the rate of postoperative adhesions.

Mechanical stress is an important component in hernia research and surgery. Our results showed, within the sublay group, a breaking force which lay in the lower range of conventional and well-tested meshes. One might get a good overview about this issue in the study published by Pott et al., where six commonly used meshes were compared by their mechanical properties. The authors concluded that there was a large variation among the tested meshes and some mesh materials seemed to be insufficiently strong for use for hernia repair. Nevertheless, the clinical experience indicates that these mesh materials are well suited for hernia surgery. It is likely that these maximum theoretical forces rarely arise in vivo, but this might explain parts of the treatment failure rate which is commonly reported [26]. This is the same for BC patches: the pure supposition of the breaking force seems to disqualify BC as hernia mesh, but in clinical reality this might play a minor part. This might not be true for the material shrinkage, even though this has to be clarified in further experiments.

The distinct loss of breaking power in the abdominal wall replacement group reflects changes of biological material in living organisms. However, two things are up to now unclear: (1) how significant is this loss of breaking power and (2) was this result the maximum of loss or is this a time-dependent process?

In the foreground of our histologic analyses were tissue integration and material-associated inflammation reactions. We could show that biocellulose is not incorporated, as might be the case in large-pore meshes, but rather, it is encapsulated. The animals in the abdominal wall replacement group showed, directly at the dorsal section of the patch, a peritoneal layer. Therefore, we assume that a kind of “neoperitoneum” was built. Histiocytic cells and conjunctive tissue were found only at the margin of the patch. We don’t think that this reaction has a clinical relevance, since the mechanical stress might lead to a proliferation reaction. Our results are congruent to the results published by Helenius et al., who found an infiltration of biocellulose when it was subcutaneously implanted [27]. Nevertheless, the material seems to be relatively inert. The main reason is that the contact surface to the surrounding tissue is small compared to the reticular structure of conventional meshes. This might not be a clinical problem, as the mesh is well incorporated through the aforementioned encapsulation. It might be possible to allow an increased infiltration with, e.g., fibroblasts, through small perforations of the mesh. However, it should be investigated if this is really necessary or not in further studies. Furthermore, we could exclude any significant fungal infection. This was an important result, since a fungal colonization is a serious complication in a clinical daily routine, one which is often difficult to treat and, in some cases, deleterious.

In principle, it is possible to modify biocellulose with several medications to treat, e.g., local infections, after direct explantation of an infected mesh. This is not only interesting for hernia repair, but also for other uses, like bypass material (in tubular form) if the cellulose is coated with heparine.

In summary, our pilot study could show the suitability of biocellulose as mesh for hernia repair. The mesh itself would be encapsulated, but showed no signs of local infections. It might be used by applying the sublay technique, as well as an abdominal wall replacement. Even if biocellulose is not a completely new material, we have shown another potential clinical application, which is worthy of consideration in the near future. However, some modifications of the material could increase the suitability of the material even further. This could be, for example, wetting with a barrier gel to prevent adhesion, or to establish pores in the mesh to increase the incorporation potential.
